# Nanozyme-assisted amplification-free CRISPR/Cas system realizes visual detection

**DOI:** 10.3389/fbioe.2023.1327498

**Published:** 2024-01-05

**Authors:** Yuan Zhang, Wanpeng Yu, Man Wang, Lei Zhang, Peifeng Li

**Affiliations:** ^1^ Institute for Translational Medicine, The Affiliated Hospital of Qingdao University, Qingdao University, Qingdao, China; ^2^ Medical Collage, Qingdao University, Qingdao, China

**Keywords:** nanozymes, CRISPR/Cas system, colorimetry, fluorescence, visual detection

## Abstract

The CRISPR (clustered regularly interspaced short palindromic repeats)/Cas (CRISPR associated) system has proven to be a powerful tool for nucleic acid detection due to its inherent advantages of effective nucleic acid identification and editing capabilities, and is therefore known as the next-generation of molecular diagnostic technology. However, the detection technologies based on CRISPR/Cas systems require preamplification of target analytes; that is, target gene amplification steps through isothermal amplification or PCR before detection to increase target analyte concentrations. This creates a number of testing limitations, such as extended testing time and the need for more sophisticated testing instruments. To overcome the above limitations, various amplification-free assay strategies based on CRISPR/Cas systems have been explored as alternatives, which omit the preamplification step to increase the concentrations of the target analytes. Nanozymes play a pivotal role in enhancing the sensitivity of CRISPR-based detection, enabling visual and rapid CRISPR assays. The utilization of nanozyme exceptional enzyme-like catalytic activity holds great promise for signal amplification in both electrochemical and optical domains, encompassing strategies for electrochemical signal sensors and colorimetric signal sensors. Rather than relying on converting a single detection target analyte into multiple analytes, these methods focus on signal amplification, the main mechanism of which involves the ability to form a large number of reporter molecules or to improve the performance of the sensor. This exploitation of nanozymes for signal amplification results in the heightened sensitivity and accuracy of detection outcomes. In addition to the strategies that improve sensor performance through the application of nanozymes, additional methods are needed to achieve visual signal amplification strategies without preamplification processes. Herein, we review the strategies for improving CRISPR/Cas systems that do not require preamplification, providing a simple, intuitive and preamplification-free CRISPR/Cas system detection platform by improving in-system one-step amplification programs, or enhancing nanozyme-mediated signal amplification strategies.

## 1 Introduction

The CRISPR (clustered regularly interspaced short palindromic repeats)/Cas (CRISPR associated) system consists of a CRISPR array and CRISPR-related proteins (Cas proteins). The CRISPR array contains repeated and spaced sequences that generate CRISPR RNA (crRNA), which binds to the corresponding Cas proteins to form a ribonucleoprotein (RNP) complex that functions as a genomic DNA editor (Zhang D. et al., 2023a). The CRISPR/Cas system has proven to be a powerful tool for nucleic acid detection due to its inherent advantages of effective nucleic acid identification and editing capabilities, and is therefore known as the next-generation of molecular diagnostic technology.

Detection technologies based on CRISPR/Cas systems require preamplification of target analytes; that is, target gene amplification steps through isothermal amplification or PCR before detection to increase target analyte concentrations, such as specific high-sensitivity enzymatic reporter unlocking (SHERLOCK) and DNA endonuclease-targeted CRISPR trans reporter (DETECTR) for ultrasensitive RNA and DNA detection, respectively (Kellner et al., 2019; Mustafa and Makhawi, 2021). However, preamplification not only extends the detection time but also brings limitations to subsequent detection, such as nonspecific amplification and interference between primers. In addition, this two-step reaction system (i.e., preamplification and detection) significantly increases the risk of aerosol contamination. Some scholars have used single-tube solutions to reduce the potential for carrying contamination, but at the expense of the optimal performance of both reactions to some extent. To overcome the above limitations, various amplification-free assay strategies based on CRISPR/Cas systems have been explored as alternatives, which omit the preamplification step to increase the concentrations of the target analytes; they have the advantages of high specificity, flexibility, and short detection time (Zavvar et al., 2022). Detection target analytes have expanded from nucleic acids to other analytes, such as living bacterial cells, proteins, and metal ions (Chang and Li, 2023). Rather than relying on converting a single detection target analyte into multiple analytes, these methods focus on signal amplification, the main mechanism of which involves the ability to form a large number of reporter molecules or to improve the performance of the sensor.

Nanozymes play a pivotal role in enhancing the sensitivity of CRISPR-based detection, enabling visual and rapid CRISPR assays. The utilization of nanozyme exceptional enzyme-like catalytic activity holds great promise for signal amplification in both electrochemical and optical domains, encompassing strategies for electrochemical signal sensors and colorimetric signal sensors (Lee et al., 2022). This exploitation of nanozymes for signal amplification results in the heightened sensitivity and accuracy of detection outcomes.

In addition to the strategies that improve sensor performance through the application of nanozymes, additional methods are needed to achieve visual signal amplification strategies without preamplification processes. Herein, we further review the strategies for improving CRISPR/Cas systems that do not require preamplification, providing a simple, intuitive and preamplification-free CRISPR/Cas system detection platform by improving in-system one-step amplification programs, or enhancing nanozyme-mediated signal amplification strategies.

## 2 CRISPR/Cas system

### 2.1 The working principles of CRISPR/Cas systems

The CRISPR/Cas system is divided into 2 classes, 6 types, and 48 subtypes. In the first class of CRISPR/Cas systems, the effector complex is composed of multiple Cas proteins, including types I, III, and IV. The second class of CRISPR/Cas systems has an effector complex consisting of a single Cas protein, including types II, V, and VI. Extensive research suggests that the II-type Cas9 (Csn1), V-type Cas12a (Cpf1), Cas12b (C2c1), Cas12f (Cas14), VI-type Cas13a (C2c2), and Cas13b (C2c6) systems within the second class of CRISPR/Cas systems hold significant potential in the field of nucleic acid detection (Makarova et al., 2015; Wang M. et al., 2020b).

Based on the detection principles, nucleic acid detection technologies based on the CRISPR/Cas system can be categorized into two types: typical targeted recognition and atypical nonspecific trans-cleavage. The first type involves the CRISPR/Cas9 system guided by single-guide RNA (sgRNA), which specifically recognizes the target strand (TS) containing homologous sequences to sgRNA near the protospacer adjacent motif (PAM) and activates the nucleolytic domain of the Cas9 protein to specifically cleave the target strand and nontarget strand (NTS) (Bolotin et al., 2005). The second type includes the CRISPR/Cas12, Cas13, and Cas14 systems guided by crRNA, which specifically recognize the target strand containing sequences homologous to crRNA, either in a PAM-dependent or PAM-independent manner. These systems activate the ’specific cis-cleavage activity and nonspecific trans-cleavage activity of the Cas protein. Cas ’protein trans-cleavage activity, also known as collateral cleavage, allows for nonspecific cleavage of adjacent single-stranded nucleic acids, which is a feature widely applied in the field of nucleic acid detection (Makarova et al., 2020).

CRISPR‒Cas9, CRISPR‒Cas12a, and CRISPR‒Cas13a can all be used for the detection of disease-related nucleic acid markers. CRISPR‒Cas9 has the capability to recognize a broad range of double-stranded DNA sequences. However, the system has limited signal amplification capacity due to its lack of trans-cleavage activity.

CRISPR/Cas13a, in contrast, is suitable for the detection of RNA targets. Cas13 protein possesses two distinct higher eukaryote and prokaryote nucleotide-binding (HEPN) domains and functions as an RNA ribonuclease within the CRISPR/Cas system. The HEPN domain substrate for cleavage is a single-stranded RNA (ssRNA) reporting probe. Guided by crRNA, Cas13 identifies the target ssRNA sequence and activates its specific cis-cleavage activity and nonspecific trans-cleavage activity, allowing it to cleave any ssRNA (Shmakov et al., 2015; Zhao et al., 2022). Unlike Cas12, Cas13 relies on a protospacer flanking site (PFS) for target recognition and cleavage, which reduces the base requirements for Cas enzymes in the target sequence (avoiding G bases at the PFS site), expanding the range of detectable target sequences. Since RNA is susceptible to nonspecific degradation, reactions need to be conducted in a system without RNA enzymes, increasing the cost and complexity of the procedure.

CRISPR/Cas12a can be activated by both dsDNA and ssDNA targets, leading to its trans-cleavage activity. When the target is dsDNA, guided by crRNA, Cas12a recognizes the target sequence downstream of a PAM site rich in thymidine nucleotides (5′-TTTN-3′, where N represents any base) (Koonin and Makarova, 2019; Qian et al., 2019; Lv et al., 2021). The system first activates specific cis-cleavage activity and cleaves the target dsDNA sequence using the RuvC domain. Following cis-cleavage, the Cas12a-crRNA dsDNA ternary complex releases the cleavage product at the distal end of the PAM site, undergoes a conformational change, and activates the nonspecific trans-cleavage activity of CAs12a (cleaving any ssDNA). When the target is ssDNA, guided by crRNA, Cas12a recognizes the target ssDNA sequence in a PAM-independent manner and directly activates nonspecific trans-cleavage activity. It is important to note that different Cas12 proteins have different preferences for various target types. Cas12a exhibits high trans-cleavage activity when bound to dsDNA targets, while Cas12b shows high trans-cleavage activity when bound to ssDNA targets (Chen et al., 2018; Li et al., 2018). Activated CRISPR/Cas12a can cleave reporter probes at a rate of thousands of times per second, amplifying the target signal. Additionally, its substrate for cleavage is ssDNA reporter probes, which are more stable and less susceptible to nonspecific degradation than ssRNA reporter probes. Therefore, there is no need for additional precautions or a clean environment to avoid the influences of RNA enzymes during the procedure. Furthermore, through signal transduction and amplification, CRISPR/Cas12a can be used for RNA target detection. Benefiting from these advantages, CRISPR/Cas12a is continually explored for applications in the field of molecular diagnostics and holds promise as an efficient molecular diagnostic tool. While using a single CRISPR/Cas12a for target nucleic acid detection achieves sensitivity at the subnanomolar (nM) level, combining CRISPR/Cas12a with isothermal nucleic acid amplification methods greatly enhances its sensitivity, enabling attomolar (aM)-level target nucleic acid detection. This level of sensitivity is comparable to the gold standard method PCR. Additionally, since CRISPR/Cas12a can specifically recognize amplification products, its use in conjunction with isothermal nucleic acid amplification methods mitigates interference from nonspecific amplification products, thereby enhancing its specificity (Tian et al., 2022). Moreover, the signal amplification of nucleic acid markers based on CRISPR/Cas12a can be achieved at a constant temperature (37°C), eliminating the need for expensive and precise instruments during amplification and making nucleic acid marker detection increasingly accessible. The nucleic acid marker detection method based on CRISPR‒Cas12a can utilize various signal output modes, such as fluorescence signals, electrochemical signals, and colorimetric signals. The key to using different signals for detection lies in the design of ssDNA reporter probes. By labeling ssDNA reporter probes differently, various instruments and devices can be employed to detect the corresponding signals.

Cas14, similar to Cas12, belongs to the Class 2 Type V CRISPR/Cas system and shares the characteristic RuvC nuclease domain of Type V Cas proteins (Takeda et al., 2021). However, unlike Cas12, Cas14 can only recognize one type of target: ssDNA. Additionally, guided by the crRNA–tracrRNA complex, Cas14 recognizes the target ssDNA sequence in a PAM-independent manner and activates its specific cis-cleavage activity and nonspecific trans-cleavage activity (cleaving any ssDNA) (Karvelis et al., 2020). Cas14 possesses the ability to accurately detect DNA single-nucleotide polymorphisms (SNPs) (Hillary and Ceasar, 2023).

### 2.2 Current challenges of the CRISPR/Cas system

Although CRISPR/Cas detection technology is rapidly advancing, its practical application involves numerous challenges. First, due to the mismatch tolerance of Cas proteins, the inherent off-target effects of the CRISPR/Cas system result in nonspecific binding, compromising the method’s specificity. Future improvements may involve developing Cas protein variants, such as SpCas9-HF1 and HypaCas9, to reduce off-target nonspecific cleavage and enhance method specificity (Kleinstiver et al., 2016; Ikeda et al., 2019). Second, compared to real-time fluorescence quantitative PCR, which is highly versatile, the PAM sequence dependency of Cas12 proteins during target sequence recognition limits the detectable target sequence range in pathogen detection. Future studies could focus on developing Cas protein variants (e.g., Cas14) independent of PAM sequences or replacing typical PAM sequences (TTTV) with suboptimal PAM sequences (e.g., VTTV, TTVV) to expand the detection range and achieve universal applicability. Third, various Cas proteins have distinct limitations due to their characteristics. For instance, Cas9 proteins can only target and cleave the target sequence without the signal amplification effect of Cas12 proteins, and complicated experiments are needed, as well as additional steps to enhance the sensitivity of nucleic acid detection. Exploring unknown Cas systems or artificially modifying known Cas proteins could address current limitations in nucleic acid detection. Moreover, nucleic acid detection based on Cas12 and Cas13 proteins relies on their nonspecific cleavage activity, hindering the CRISPR/Cas system’s ability to perform multiplex detection of various target sequences in a single system, unlike real-time fluorescence quantitative PCR. Addressing this challenge involves leveraging Cas protein preferences for base editing and combining different Cas proteins, such as LwaCas13a, to design orthogonal experiments and simultaneously detect multiple targets (Gootenberg et al., 2018).

## 3 Nanozyme-assisted CRISPR/Cas system

Nanozymes are a category of nanomaterials with enzymatic catalytic activity (Zandieh and Liu, 2023). Nanozymes combine the advantages of biological enzymes and nanomaterials, exhibiting high catalytic performance and exceptional stability (Mou et al., 2022). Since the first report of Fe_3_O_4_ nanozymes in 2007, various nanomaterials with multienzyme activity have been discovered (Gao et al., 2007). Depending on their catalytic functions, nanozymes can be categorized into oxidative–reductive enzymes, hydrolytic enzymes, ligase enzymes, and isomerase enzymes. Oxidative–reductive enzyme-like nanozymes account for over 90% of these four types of catalytic nanozymes. These nanozymes feature enzyme activities similar to oxidase (OXD), peroxidase (POD), catalase (CAT), superoxide dismutase (SOD), glucose oxidase (GOx), and glutathione peroxidase (GPx) (Zhao et al., 2021; Xu et al., 2022; Zhang et al., 2023c). In the field of analytical sensing, commonly used nanozymes include POD-like, CAT-like, OXD-like, and GOx-like nanozymes. Nanozymes offer several advantages over natural enzymes, including enzyme-like reaction kinetics, high catalytic efficiency, and tolerance to environmental changes relative to natural enzymes. Nanozymes can be modified to have a large surface area-to-volume ratio, thereby enhancing their catalytic activity. Moreover, nanozymes are more cost-effective, can be produced in larger quantities, and exhibit more robust, stable, and tunable activity than natural enzymes. Due to their unique enzyme catalytic properties and achievable colorimetric reactions, nanozymes have gained widespread attention for the sensitive detection of biomolecules under complex physiological conditions using colorimetry, fluorescence, and electrochemical detection methods (Niu et al., 2022; Ren et al., 2022; Kurup and Ahmed, 2023). As a result, nanozymes as signal labels in biomolecular detection can circumvent many limitations associated with natural enzymes.

### 3.1 Regulation of nanozyme activity

A unique advantage of nanozymes lies in their modifiability, as their catalytic capabilities can be extended beyond classical enzymes through various approaches.

First, the catalytic activity can be promoted by adjusting the nanozyme size. Changes in nanoparticle size directly impact the active sites on the surface, influencing catalytic activity. Generally, catalytic activity increases as nanoparticle size decreases. Catalytic activity can be regulated through synthetically controlling the size of nanozymes (Xi et al., 2020). Second, the catalytic activity of nanozymes can be influenced by changes in oxidation states. The surface oxidation states of nanozymes are closely related to catalytic activity, and different states and their proportions lead to variations in catalytic activity and types (Sims et al., 2019). Furthermore, catalytic activity can be facilitated by controlling the crystal facets of nanozymes. Regulating crystal surface exposure is a highly effective strategy in catalytic activity control. The morphology of material is commonly adjusted to achieve surface control, exposing different crystal facets. Different facets exhibit distinct atomic arrangements and dangling bond quantities, further impacting the rate and selectivity of material enzyme catalytic reactions (Mu et al., 2014). Surface modification of nanozymes can enhance the catalytic activity. As catalytic reactions generally occur at the material’s surface, modifying the surface can regulate properties such as surface charge, active site exposure, and substrate binding affinity. Modification types include group modification, ion modification, and polymer modification. Surface modification provides a flexible and controllable strategy for regulating nanozyme catalytic activity (Fan et al., 2016). Additionally, the catalytic activity can be promoted by altering the composition of nanozymes. Compared to single-component materials, multicomponent nanomaterials exhibit synergistic effects, enhancing material performance. Enzyme activity can be regulated by changing the proportions of various component materials in nanozymes. Furthermore, heteroatom doping enhances catalytic performance by adjusting conductivity or carrier density (Chen and Song, 2022). For many reactions, single-metal-based catalysts cannot achieve high selectivity and efficiency. The synergistic effect of different metals alters the nanozyme structure, resulting in significantly enhanced catalytic activity for multimetal-based nanozymes (Yan et al., 2019).

### 3.2 The detection mechanism of nanozyme-assisted CRISPR/Cas system

**FIGURE 1:**
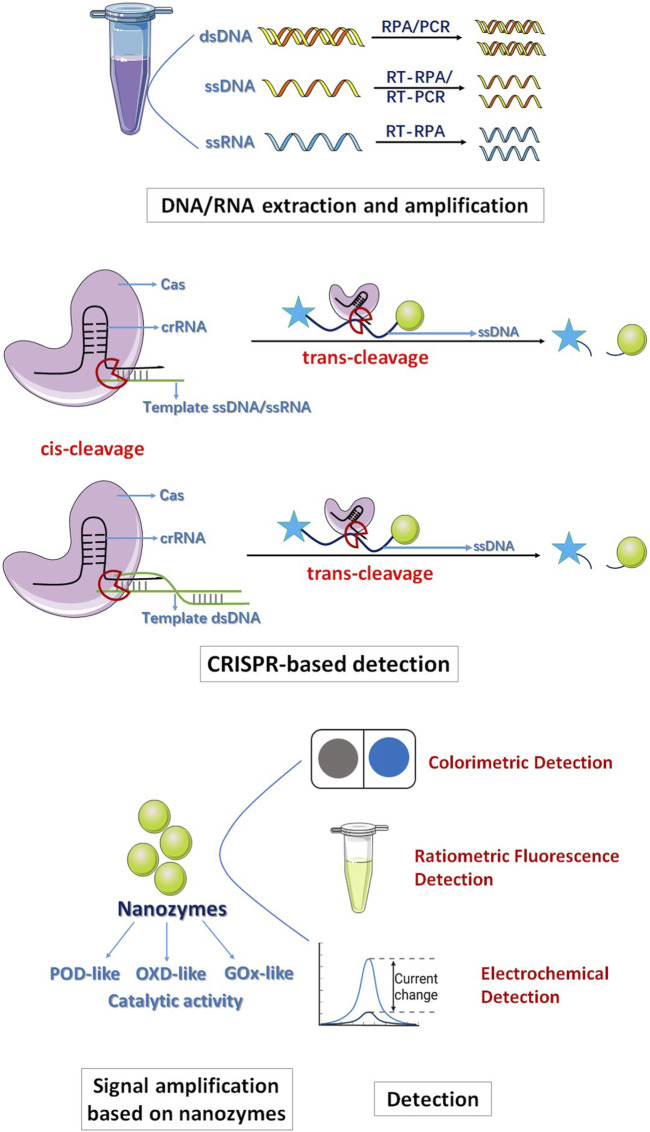
The schematic for nanozyme-assisted CRISPR/Cas System.

In general, the entire detection process consists of four steps. First, DNA/RNA extraction and amplification are conducted. DNA/RNA is extracted through the use of a reagent kit, followed by amplification via recombinase polymerase amplification (RPA) and reverse transcription-RPA (RT-RPA) methods. This approach facilitates rapid target DNA/RNA amplification under ambient temperature conditions. Second, CRISPR-based detection is employed. After the identification of the target sequence, crRNA complexes exhibit specific cleavage activity toward ssDNA/dsDNA/ssRNA (cis-cleavage), furthermore, the activated Cas exhibit indiscriminately cleavage activity (trans-cleavage) to ssDNA. Notably, nonspecific ssDNA trans-cleavage occurs at a rate of approximately 17 times per second. This trans-cleavage presents a significant strategy for enhancing the efficiency, sensitivity, and precision of biomolecular detection. Third, signal amplification is achieved through the utilization of nanozymes. Integration of the exceptional enzyme-like catalytic activity of nanozymes results in highly efficient and sensitive CRISPR-based detection outcomes. Last, the detection results are output through various techniques, including electrochemical signals and colorimetric signals (Figure 1) (Dai et al., 2020). For example, Chen et al. (2023) developed a colorimetric strategy based on Au-Fe_3_O_4_ nanozymes combined with CRISPR/Cas12a to diagnose antibiotic resistance. When the CRISPR/Cas12a system identifies the target gene, the Cas12a crRNA complex exhibits cis-specific cleavage activity to dsDNA, releases the cleavage product and changes conformation; subsequently, the trans-cleavage activity of Cas12a is activated to perform nonspecific cleavage of ssDNA and releases Au-Fe_3_O_4_ nanozymes. The Au-Fe_3_O_4_ nanozymes exhibit POD-like activity and can oxidize 3,3,5,5-tetramethylbenzidine (TMB) under mild conditions, changing the color from transparent to blue; thus, the nanozyme-assisted CRISPR/Cas12a system makes detection simple and visible (Chen et al., 2023). Wu et al. (2022) constructed a colorimetric strategy of the CRISPR/Cas12a system assisted by MnO_2_ nanozymes to detect SARS-CoV-2. Due to the excellent OXD-like activity of MnO_2_, the MnO_2_ nanorods connected with streptavidin-coated magnetic beads obtained by magnetic field separation can have a strong oxidation effect on TMB, and the color changes from light yellow to blue. However, when the target nucleic acid of SARS-CoV-2 is present, the trans-cleavage activity of Cas12a is activated, and the ssDNA between MnO_2_ nanorods and MBs is cleaved. After magnetic field separation, when the number of MnO_2_ nanorods bound to MBs is reduced, the catalytic reaction is weakened, resulting in a visible color reduction of the TMB solution. Therefore, the detection of SARS-CoV-2 is quantitatively observed by visual or spectrophotometric methods. In addition, the MnO_2_ nanozyme-assisted CRISPR/Cas system is simpler than the HRP-catalyzed TMB-H_2_O_2_ system because it does not require H_2_O_2_ and the nanozymes are very robust. Therefore, visual and efficient detection of target nucleic acids can be achieved. However, preamplification of target RNA with reverse transcription-recombinase polymerase amplification (RT-RPA) is needed before detection of SARS-CoV-2, thus limiting its convenience (Wu et al., 2022).

The application of nanozymes can improve the performance of sensors and assist CRISPR/Cas systems in achieving simple, visible and highly sensitive detection. Nanozymes can replace natural enzymes to label recognition molecules, such as antibodies and DNA, to prepare colorimetric sensors or ratio fluorescence sensors, which have better stability and lower cost than natural enzymes (Ye et al., 2021; Chi et al., 2023). The signal output of the nanozyme-based colorimetric analysis mainly depends on the catalytic oxidation of the substrate by the nanozymes; therefore, the colorless substrate, such as TMB, ABTS, and OPD, can be oxidized into oxidation products with specific colors and absorption spectra to generate colorimetric output signals and achieve simple and visual detection (Wang et al., 2019; Ding et al., 2021; Fu et al., 2021; Huang et al., 2022). In addition, a cascade catalytic sensing system can be constructed by combining nanozymes with various natural enzymes, such as GOx, choline oxidase, and cholesterol oxidase. For example, Broto et al. designed a novel biosensor based on CRISPR/Cas systems combined with a nanozyme-linked immunosorbent assay (NLISA), called CrisprZyme, for the detection of noncoding RNA biomarkers for myocardial infarction. Unlike traditional CRISPR diagnostic methods, CrisprZyme can perform quantitative and colorimetric readings of Cas13-mediated RNA detection at room temperature by catalyzing metal nanoparticles. The scholars used bimetallic nanozymes composed of platinum and gold (Pt@Au) in combination with Cas13 enzymes, thereby significantly improving the sensitivity of RNA detection. In this method, the peroxidase-like activity of the Pt@Au nanozyme can increase the signal strength so that the diagnostic process does not need to rely on RNA signal amplification and a series of complex diagnostic steps, such as temperature control. A master mixture containing *wdei Leptothrix* Cas13 (LwaCas13a), gRNA, and reporter RNA is mixed with target RNA to trigger the cleavage of reporter RNA labeled with FAM and biotin. The reaction products are then added to NLISA to quantify the amount of reporter RNA that is cleaved. Cleavage of the reporting RNA occurs in the presence of the target analytes, preventing the binding between the reporting RNA-mediated anti-FAM antibody and streptavidin-functionalized nanozyme, thereby reducing the binding of the nanozyme. The reduction in the number of nanozymes reduces the catalytic activity and oxidation level of the chromogenic substrate. Thus, the presence of the target analytes inhibits the development of color, while its absence increases the color change initiated by the nanozyme. Finally, the limit of detection (LOD) of CrisprZyme reaches 7.88 ± 3.21 pM, and when reacting at room temperature, the LOD of CrisprZyme is similar. Furthermore, when LbuCas13a is used instead of LwaCas13a, the LOD can be increasingly accurate to the femtomolar level (fM, 10^−15^ mol), which greatly improves the detection sensitivity. The study shows that CrisprZyme readily adapts to readings based on lateral flow and different Cas enzymes and is able to sense noncoding RNAs, including microRNAs, long noncoding RNAs, and circular RNAs (Broto et al., 2022).

The ratiometric fluorescence detection based on nanozymes quantitatively analyzes the target by using two different fluorescence emission wavelengths and recording the ratio of their emission intensity (Wang et al., 2019; Fu et al., 2022). This kind of fluorescent probe is generally designed to have two or more fluorescence emissions through the interaction of energy transfer, charge transfer, proton transfer, and chemical reaction in the presence of the target object to change the ratio of the fluorescent probe. The ratiometric fluorescence probe based on nanozymes can avoid the interference of background fluorescence and can greatly improve the sensitivity and accuracy. In some designs, the ratiometric fluorescence probe can show obvious color changes and realize naked eye detection. For example, Miao et al. developed a CeO_2_ nanozyme-based NLISA combined with graphitic carbon nitride quantum dots (g-C3N4 QDs) to achieve dual-modal colorimetric and ratiometric fluorescent detection, thus affording reliable and sensitive detection of cTnI. By using the good peroxide-like activity of the CeO_2_ nanozyme, OPD can be catalyzed to oxOPD effectively, and a new maximum emission peak can be generated at 578 nm. The g-C_3_N_4_ QDs are added as a reference fluorescence signal source. Therefore, oxOPD can be effectively immobilized on the surface of g-C_3_N_4_ QDs through hydrogen bonding and π-π stacking interactions, and the emission peak of g-C_3_N_4_ QDs at 410 nm is effectively quenched through photoinduced electron transfer (PET) effects, resulting in a ratiometric fluorescence response. In addition, since the colorless OPD is converted into orange oxOPD, visibility detection is achieved by a colorimetric assay. Thus, the dual-modal colorimetric and ratiometric fluorescence assay method enable the lowest LOD of cTnI to be 0.227 pg mL^−1^, and sensitive naked-eye readout performance is achieved even when the concentration of cTnI is as low as 1 pg mL^−1^ (Miao et al., 2019).

The electrochemical biosensor detection platform constructed based on the CRISPR/Cas system assisted by nanozymes can realize efficient and sensitive point-of-care testing (POCT) in the detection of pathogenic organisms. Electrochemical biosensors represent one of the earliest and most diverse branches within the field of biosensors. Electrochemical biosensors mainly use electrochemical principles to detect biomolecules, cells and cellular tissues, usually consisting of electrodes, biomolecular recognition layers and signal amplification and processing circuits. The biomolecular recognition layer is typically a specific biological molecule, such as an enzyme, antibody, and nucleic acid, capable of specific reactions with the target biomolecules, converting these reactions into electrical signals (Updike and Hicks, 1967; Anik et al., 2017). To date, many researchers have combined electrochemical biosensors with molecular diagnostic techniques based on the CRISPR/Cas system, creating pathogen detection platforms that utilize CRISPR/Cas and electrochemical biosensing technologies. Utilizing nanozymes to amplify electrical signals is a promising strategy. Using the exceptional catalytic activity of nanozymes can result in large initial current peaks and increasingly significant current peak changes after activating the CRISPR/Cas system. This phenomenon enables nanozyme-assisted CRISPR/Cas systems to prepare electrochemical biosensors that are highly efficient and sensitive POCT technologies. For example, Li C. et al. (2023a) designed a CRISPR/Cas14a system combined with PtPd nanoparticle-functionalized porphyrin metal–organic framework nanozymes (PtPd@PCN-224 nanozymes) as signal amplification tags to prepare an electrochemical biosensor with high sensitivity and specificity for the detection of *Burkholderia pseudomallei (B. pseudomallei)* DNA (Li C. et al., 2023a). This strategy utilizes the targeted activation of CRISPR‒Cas14a to recognize the target DNA sequence, which triggers the trans-cleavage of ssDNA for signal amplification. In the absence of target DNA, CRISPR/Cas14a is not activated, which makes phosphorylated ssDNA (P-ssDNA) unable to be cleaved at the electrode interface. Subsequently, PtPd@PCN-224 nanozymes are fixed to P-ssDNA-modified electrodes based on Zr-O-P coordination bonds. In the presence of H_2_O_2_, PtPd@PCN-224 nanozymes catalyze the degradation of H_2_O_2_, and the H_2_O_2_ reduction peak current is strong. In contrast, when the target DNA is present, the target activates CRISPR/Cas14a; P-ssDNA on the electrode interface can perform trans-cleavage activity so that PtPd@PCN-224 nanozymes cannot assemble on the electrode interface through the Zr-O-P bond, resulting in a weak reduction peak current of H_2_O_2_. In the signal-off-output mode, the electrochemical signal decreases with increasing target DNA concentration. The electrochemical biosensor has high sensitivity for detecting *B. pseudomallei* DNA due to the synergistic effect of targeted activated CRISPR/Cas14a and PtPd@PCN-224 nanozymes. The limit of detection is 12.8 aM, which enables ultrasensitive detection and has excellent specificity to distinguish nontarget bacteria (Li C. et al., 2023a).

## 4 Nanozyme-assisted preamplification-free CRISPR/Cas system

In addition to the strategies that improve sensor performance through the application of nanozymes, additional methods are needed to achieve visual signal amplification strategies without preamplification processes. Herein, we review the strategies for improving CRISPR/Cas systems that do not require preamplification.

### 4.1 Utilizing rolling circle amplification (RCA) strategies in the system to achieve preamplification-free

Padlock probes are single-stranded DNA molecules containing a region complementary to the target DNA (Thomas et al., 1999; Banér et al., 2001). When the padlock probes hybridize with their target in a liquid phase, they form a circular template through sequence-specific ligation, facilitated by DNA polymerase (such as phi29) and the presence of labeled probe DNA. This process is known as padlock rolling-circle amplification (Padlock-RCA) (Magoulopoulou et al., 2023). This technique offers high specificity and sensitivity, allowing precise identification of target sequences and minimizing false-positives. Furthermore, padlock probes can be combined with fluorescent dyes and other probes to generate fluorescence and other signals, enabling quantitative analysis of target gene expression levels. This feature gives Padlock probes a distinct advantage over PCR technology. Additionally, padlock probes possess reversibility, as their structures can be opened and closed, making them suitable for the dynamic monitoring of target gene expression levels. As a result, padlock probes hold great promise as versatile molecular tools with applications in biomedical research and clinical diagnostics.

To date, there is a significant amount of research that combines nanozymes with the CRISPR/Cas12a system and Padlock-RCA technology, establishing a novel colorimetric assay. This approach achieves high sensitivity, specificity, ease of operation, and visual detection, providing a new avenue for POCT in the field of bioassays.

**FIGURE 2:**
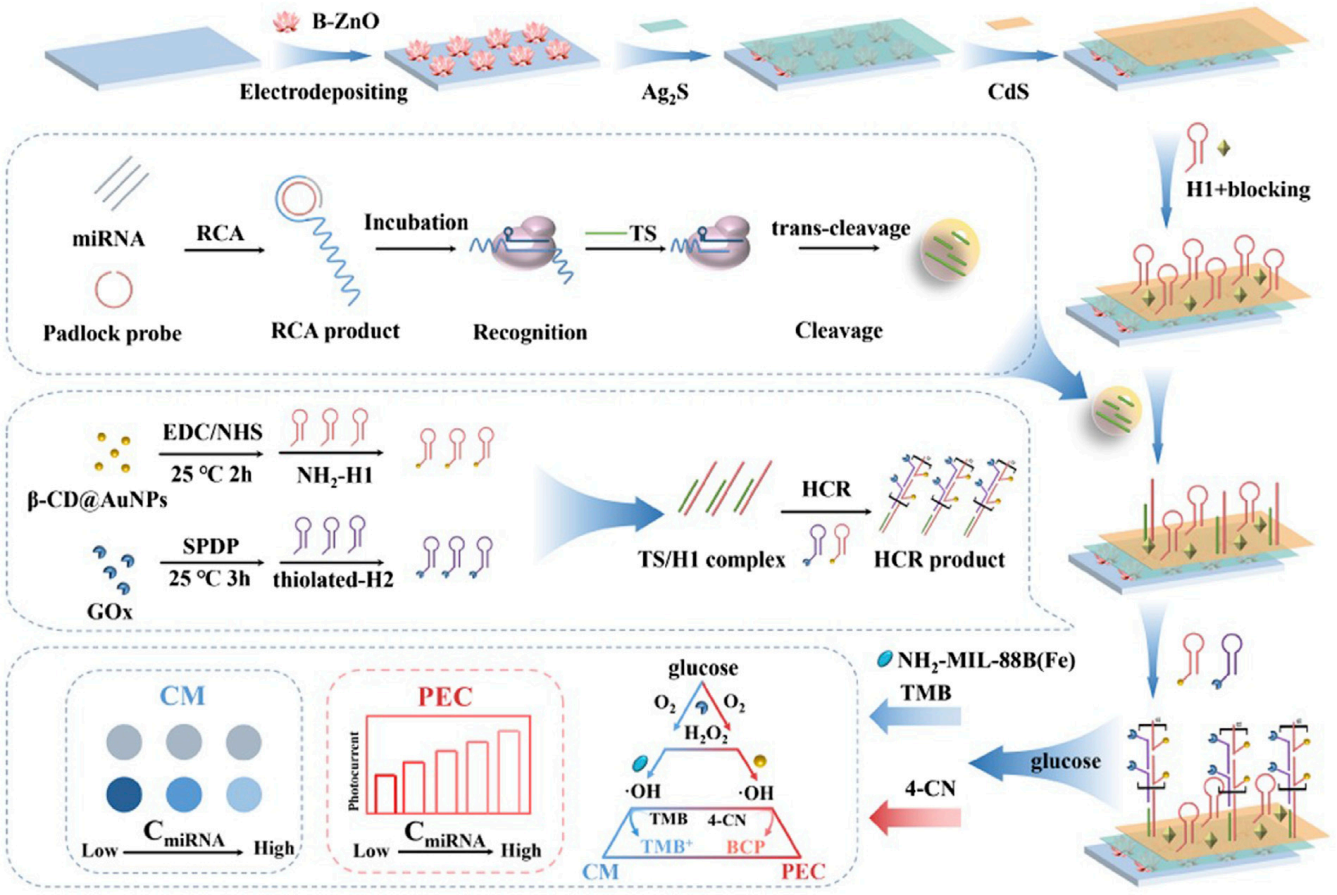
Utilizing rolling circle amplification (RCA) strategies in the system to achieve preamplification-free. The multi-amplification circuit (RCA-Cas12a-HCR) was constructed to provide the accurate and programmable detection of miRNAs. Adapted with permission from Shen et al. (2022), copyright 2022, Elsevier.

For example, Mu et al. (2023) combined Ag/Pt nanocluster nanozymes and CRISPR/Cas 12a systems with Padlock-RCA technology to establish a novel highly sensitive and specific visual sensor for the detection of carcinoembryonic antigen (CEA). The researchers connect the DNA template chain (T-DNA) to Ag/Pt bimetallic nanoclusters with peroxidase-like catalytic activity and synthesize DNA-Ag/Pt bimetallic nanoclusters as signal reporters. Then, the padlock probe (P-DNA) is designed, including the recognition region of the primer chain (C-DNA), the complementary sequence of the active chain of Cas12a/crRNA (activation chain sequence), and the complementary sequence region of the recognition site for Nb.BbvCI. When CEA is present, the target analytes specifically bind to the aptamer (A-DNA) in the DNA double-stranded complex, releasing the primer chain (C-DNA). P-DNA is complementary to C-DNA and is cycled to form an RCA template (loop) under the action of T4 DNA ligase. Then, under the action of phi29 DNA polymerase, the RCA reaction is initiated to produce a long DNA amplification product with multiple repeats consistent with the activation chain sequence. Finally, under the action of Nb. BbvCI (endonuclease), a large number of Cas12a/crRNA activation sequences and C-DNA are generated. The resulting C-DNA reactivates a new round of RCA, while the activator can activate the trans-cleavage activity of Cas12a/crRNA, further degrading the DNA probe sequence of DNA-Ag/Pt nanoclusters, and the absorbance of the system at 652 nm is significantly reduced. However, in the absence of the target CEA, template cyclization of the ring cannot be achieved, and P-DNA is gradually degraded by phi29 DNA polymerase with 3′-5′s single-stranded DNA extranuclear decomposition activity, resulting in a lack of RCA product and the inability to activate the trans-cleavage activity of Cas12a/crRNA. At this time, adding DNA-Ag/Pt nanoclusters with peroxidase-like catalytic activity can catalyze H_2_O_2_ to oxidize TMB to blue substances, and the absorbance increases sharply at 652 nm. Signal amplification by the combination of nanase, CRISPR/Cas12a system and Padlock-RCA technology significantly improves the sensitivity of CEA detection. The linear range can reach 2.5 pg/mL∼2.0 ng/mL, and the detection limit is 0.94 pg/mL (S/N = 3). Therefore, this method can realize CEA visual detection by adding color differences before and after the target analytes (Mu et al., 2023). Shen et al. combined nanozymes, the CRISPR/Cas12a system and RCA technology to construct a biosensor based on photoelectric chemistry (PEC) and colorimetry (CM) for the efficient and specific detection of miRNA (Figure 2; Table 1) (Shen et al., 2022). The device first captures miRNAs via a padlock probe, and with the assistance of T4 DNA ligase and phi29 DNA polymerase, the miRNAs complement the circular padlock probe to trigger an RCA reaction that converts miRNAs into long ssDNA with large repeat regions. The generated ssDNA can be specifically recognized by Cas12a, activate the trans-cleavage activity of Cas12a, and cleave the TS (trigger strand) needed for the HCR reaction, thus reducing the TS that can participate in the HCR reaction. As a result, relatively few β-CD@AuNPs-H1 and GOx-H2 probes are assembled on the surface of the photoelectrode via the HCR reaction and respond via photoelectrochemistry (PEC), resulting in a weak change in photocurrent. GOx-H2 is coupled with NH2-MIL-88B(Fe) nanozymes to oxidize TMB through a cascade reaction that allows the colorimetry (CM) response to produce visible color changes. This sensor combines GOx with β-CD@AuNPs and NH2-MIL-88B(Fe) nanozymes to achieve a PEC-CM signal in a double cascade system. Therefore, the greater the miRNA content is, the lower the TS content in the reaction, and the fewer TSs that can participate in the HCR reaction, resulting in more significant signal changes. The detection of miRNA is convenient and sensitive. The final detection limits are 0.3 fM (PEC) and 0.5 fM (CM). The application of nanozymes and Padlock-RCA technology provides a novel solution for the CRISPR/Cas12a system to be applied in POCT and the early diagnosis of diseases (Table 1) (Shen et al., 2022).

**TABLE 1:** Summary of nanozyme-assisted preamplification-free CRISPR/Cas system detection strategies.

Technology	Cas type	Nanozyme type	Signal read-out	Targets and sample type	LOD	References
Combined Padlock-Rolling circle amplification (RCA)	Cas12a	Peroxidase activity	Colorimetry	Carcinoembryonic antigen (CEA) in human serum	0.94 pg/mL	Mu et al. (2023)
Combined Padlock-RCA	Cas12a	Peroxidase activity	photoelectric chemistry and colorimetry	miRNA-21 in the serum	0.3 fM or 0.5 fM	Shen et al. (2022)
DNA-modifying nanozyme	Cas12a	Peroxidase activity	Colorimetry	hepatitis B virus DNA in spiked human serum	0.5 pM	Tao et al. (2022)
Combined immunomagnetic bead technique (IMBT)	Cas12a	Oxidase activity	Colorimetry	Pb^2+^ in food samples	0.54 nM	Xu et al. (2023)
Combined IMBT	Cas12a	Peroxidase activity	Photoelectrochemical (PEC)	Interleukin-4 (IL-4) in artificial cerebrospinal fluid	0.32 fg/mL	Zhang et al. (2023b)
Dopamine-modified probe	Cas12a	Peroxidase activity	Electrochemiluminescence (ECL)	exomiRNA-155 in serum samples of breast cancer	273.20 aM	Shen et al. (2023)
Dopamine-modified probe	Cas12a	Oxidase activity	ECL	cytochrome c oxidase subunit III (COX III) gene in human urine samples	0.18 pM	Liu et al. (2022)

### 4.2 DNA modification enhances the catalytic activity of nanozymes to achieve signal amplification in the CRISPR/Cas system

The modification of nanozymes by aptamers (short strand DNA/dsDNA/ssDNA) can enhance the activity of nanozymes and enhance the sensitivity of detection. DNA can be used as an enzyme modulator for the highly controllable regulation of the catalytic behaviors of nanozymes to further promote enzyme-like activity. Studies have shown that DNA modifications can alter the inherent properties of nanoparticles (e.g., plasmon coupling and optical absorption) and enhance their recognition of various analytes. The catalytic activity of nanozymes can be significantly enhanced by the modification of coiled single-stranded DNA and hairpin DNA. Therefore, the modification of DNA with different structures has a significant effect on the catalytic activity of nanozymes.

**FIGURE 3:**
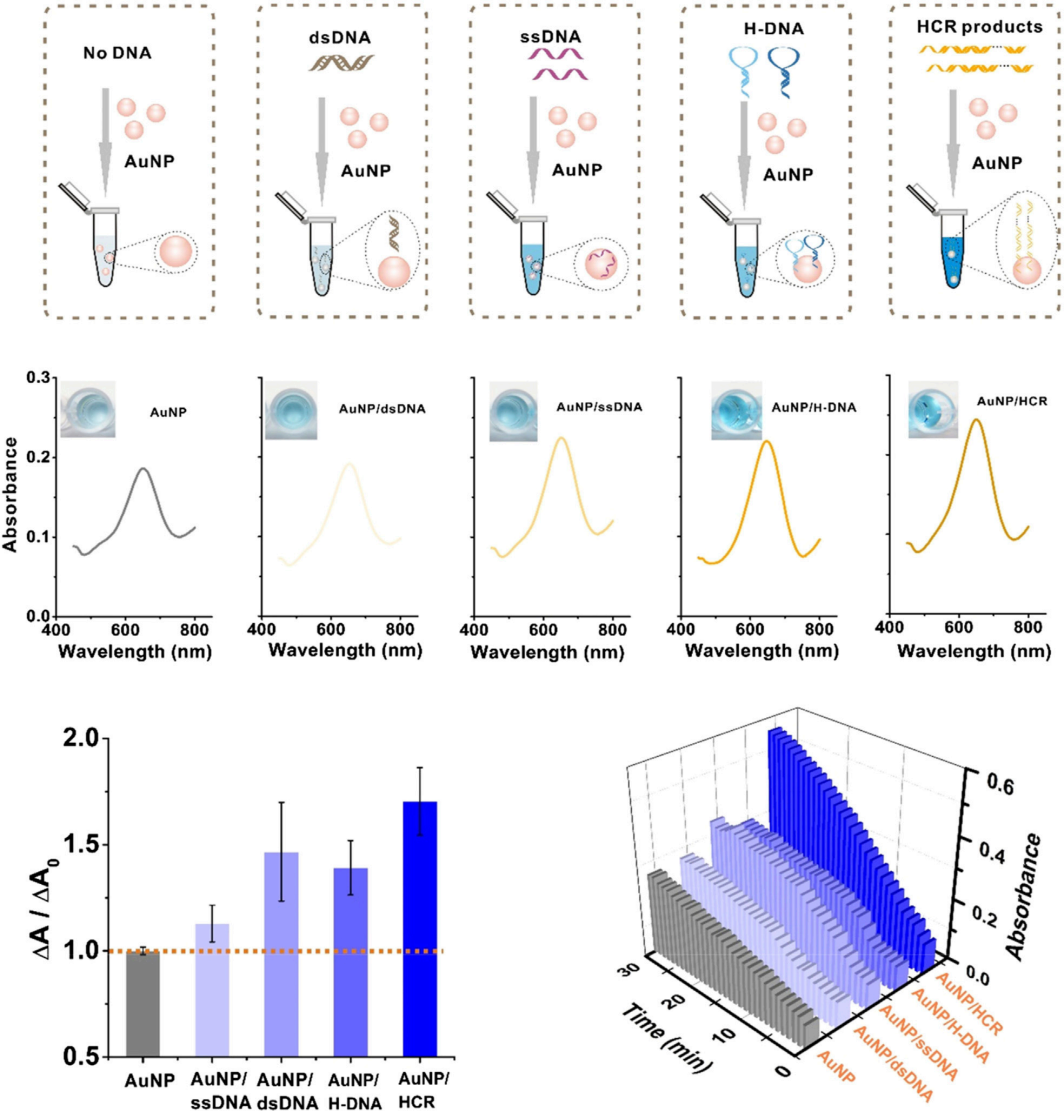
DNA modification enhances the catalytic activity of nanozymes to achieve signal amplification in the CRISPR/Cas system. Modifying Fe_3_O_4_ nanozymes with ssDNA, dsDNA, H-DNA and HCR products have different effects on the catalytic activity of Fe_3_O_4_ nanozymes. Adapted with permission from Zeng et al. (2019), copyright 2019, American Chemical Society.

For example, Zeng et al. (2019) showed that modifying Fe_3_O_4_ nanozymes with the single-stranded DNA (ssDNA), double-stranded DNA (dsDNA), hairpin DNA (H-DNA) and long dsDNA of hybridization chain reaction (HCR) products have different effects on the catalytic activity of Fe_3_O_4_ nanozymes; the strengths of the effects are in the following order: HCR product >H-DNA>ssDNA>dsDNA (Figure 3). dsDNA with complementary double strands weakly interact with the surfaces of Fe_3_O_4_ nanoparticles (NPs), while ssDNA with 24 exposed bases has a high affinity with the surfaces of Fe_3_O_4_ NPs, indicating that ssDNA-modified Fe_3_O_4_ NPs have a higher enzymatic activity than dsDNA-modified Fe_3_O_4_ NPs. H-DNA with a secondary stem‒loop structure and a 6-mer ssDNA tail has a relatively strong binding with Fe_3_O_4_ nanozymes and great freedom to interact with the substrate. HCR products mimic a one-dimensional, linear, and long dsDNA structure composed of hundreds of repeated units with 24-mer ssDNA sticky ends, inducing the strongest affinity for Fe_3_O_4_ NPs; thus, HCR-modified Fe_3_O_4_ nanozymes have the strongest enzymatic activity. Furthermore, HCR products, due to carrying the most negative charge, exhibit the strongest binding capability for TMB oxidation. Consequently, HCR products result in the highest enhancement of activity on Fe_3_O_4_ NPs. Conversely, H-DNA and ssDNA carry relatively few negative charges, thus contributing to a moderate enhancement of the peroxidase-like activity on Fe_3_O_4_ NPs. However, dsDNA, which has the least negative charge, has such weak binding interactions that its enhancement effect on Fe_3_O_4_ NPs can be considered negligible (Zeng et al., 2019). Liu et al. showed that the modification of long DNA, especially DNA containing polycytosine (C), can significantly enhance the catalytic activity of Fe_3_O_4_ nanozymes. The order of reaction kinetics is C > G > T > A. The negatively charged DNA phosphate backbone and bases can enhance the binding of Fe_3_O_4_ nanoparticles to TMB, thus promoting oxidation in the presence of H_2_O_2_. The DNA bases promote substrate binding by hydrogen bonding to the amino group of TMB; conversely, the nuclear bases may interact with the benzene ring of TMB by π-π stacking. Zhao et al. found that the catalytic activity of MoS_2_ nanosheets (NSs) modified by negatively charged polymers can significantly improve the catalytic activity of MoS2 NSs, among which ssDNA is the most effective promoter. The enhanced catalytic activity of MoS_2_ NSs modified by DNA is mainly due to the increased affinity of DNA/MoS_2_ NSs for the peroxidase substrate TMB rather than the production of hydroxyl radicals. ssDNA containing a negative charge can be adsorbed to the surface of MoS_2_ NSs by van der Waals forces, significantly increasing the electrostatic attraction between MoS_2_ NSs and TMB. The increase in affinity can promote contact between the active site of MoS_2_ NSs and the substrate TMB and further accelerate the transfer of electrons from the lone pair electrons in the TMB amino group to MoS_2_ NSs. Subsequently, the electron density and mobility characteristics of MoS_2_ NSs are enhanced, resulting in an accelerated transfer of electrons from MoS_2_ NSs to H_2_O_2_. Therefore, the addition of ssDNA significantly accelerates the electron transfer process of TMB to H_2_O_2_ catalyzed by MoS_2_ NSs, thereby improving the catalytic activity of MoS_2_ NSs. The catalytic activity of ssDNA-modified MoS_2_ NSs follows the trend of G ≈ T > A > C > no DNA. Therefore, after ssDNA modification, the peroxisase-like activity of MoS_2_ NSs is significantly increased by 4.3 times (Zhao et al., 2020).

Therefore, DNA-modified nanozymes containing additional negative charges can promote contact between the active site of the nanozymes and the substrate TMB through the electrostatic attraction of negative charges so that the catalytic activity of the nanozymes is significantly improved. By modifying a negatively charged DNA (ssDNA) strand probe on the surface of the nanozymes, the activated CRISPR/Cas system can promote the catalytic activity of the nanozymes through nonspecific trans-cleavage of the DNA probe, and the enhanced catalytic activity significantly improves the sensitivity of the CRISPR/Cas system for detection. For example, Tao et al. established a colorimetric biosensor based on a nanozyme-assisted CRISPR/Cas12a system to detect HBV DNA by regulating the catalytic behaviors of Ag/Pt NP-modified Ti3C2Tx MXene nanostructures with probe DNA. This study confirmed that polynucleotide ssDNA can act as a linker to modify Ag/Pt NPs on Ti3C2Tx MXenes and as a nanozyme mediator to achieve the highly controllable regulation of the catalytic behaviors of Ti3C2Tx MXenes to improve their peroxidase-like enzymes. Importantly, the catalytic activities of polynucleotide-modified Ti3C2Tx MXenes are different in the order of polyG > polyT > polyA > polyC > no DNA. The catalytic activity of the MXene-polyG DNA nanocomplex is approximately 22 times higher than that of the bare MXene nanosheets. When HBV targets are present, the trans-cleavage ability of the Cas12a enzyme can be activated to degrade the DNA probe, thereby inhibiting the adsorption of DNA metallization and the enzyme activity enhancer DNA to MXene, minimizing catalytic activity and significantly changing color visible to the naked eye. A combination of the CRISPR/Cas12a system and MXene-DNA-Ag/Pt nanocomplex as enzymatic mimics achieves the first and second signal amplification providing a novel and intuitive method for the accurate and sensitive analysis of HBV DNA with the naked eye, without the need for the amplification of target DNA (Table 1) (Tao et al., 2022).

Therefore, the oligonucleotides (such as aptamers) modifiability of nanozymes and the visualization of detection results may have the potential to be a simple and low-cost signaling output for CRISPR diagnostics.

### 4.3 Immunomagnetic bead techniques assist in amplifying the signal of nanozymes

The immunomagnetic bead technique (IMBT) is a detection and separation technology based on immunological principles (Oksvold et al., 2015; Pan et al., 2016). IMBT is a biological technique that employs superparamagnetic magnetic microspheres, also known as immunomagnetic beads, as carriers to capture and enrich target substances. Typically, the surfaces of immunomagnetic beads are coated with biological antibodies or other ligands. These beads are mixed and incubated with samples containing the target of interest. During this process, the target substance binds to the biological antibodies or ligands on the magnetic beads, forming a bead-biological antibody (or another ligand)-target complex. Subsequently, under the influence of an applied magnetic field and due to the magnetic responsiveness of the beads, the complex is gathered. This phenomenon allows for the efficient separation of the target substance from environmental materials, eliminating external influences. Immunomagnetic separation (IMS) offers several advantages, including a large bead surface area, increasing the chances of ligand‒receptor contact and enhancing detection sensitivity. Additionally, magnetic beads exhibit excellent magnetic responsiveness, resulting in fast separation, straightforward operation, high efficiency, and no damage to the target substances. Furthermore, magnetic beads serve as a fluid‒solid phase carrier, facilitating effective reactions. These advantages underpin the promising and widespread applications of IMBT. To date, immunomagnetic separation finds excellent use in various applications, such as the separation and enrichment of microorganisms, nucleic acid extraction, protein purification, and cell sorting. For example, Du et al. used IMBT as a signal amplification mediator and combined a quantum dot probe with the CRISPR/Cas12a system to realize the visual detection of African swine fever virus. The method first binds biotinated ssDNA to streptavidin-modified magnetic beads, and in the presence of the target analytes, the target analytes identify with CRISPR/Cas12a and initiate their trans-cleavage activity, causing the biotinylated ssDNA probe to cleave. Subsequently, quantum dot-modified ssDNA is added, which can hybridize with the uncut probe on the magnetic bead. After magnetic field separation of the magnetic beads hybridized with the quantum dot reporter, a portable ultraviolet flashlight is used to irradiate the collected supernatant to achieve simple and convenient instant detection. The LODs of African swine fever virus obtained in buffer and pig plasma are approximately 0.5 and 1.25 nM, respectively. Compared with the reporter using fluorophores and quenched groups, the introduction of magnetic beads and quantum dots as reporters can effectively reduce the influences of background signals and improve the sensitivity of virus detection (Bao et al., 2020). Qin et al. used the magnetic bead−ssDNA–Pt NP (BDNP) conjugate as a quantitative reading reporter for the CRISPR/Cas12a system and combined it with a magnet-assisted volumetric bar-chart chip (MAV-chip) to build a visual, instrument-free quantitative detection platform for single nucleotide polymorphisms (SNPs). During the MAV-chip assay, when the target analytes are present, CRISPR/Cas12a nonspecifically cleaves BDNPs to release Pt NPs from the magnetic bead and subsequently uses a magnetic field to separate the Pt NPs through the magnetic bead. After incubating the chip at 37°C for 1 h, by moving the platform upward, Pt NPs and H_2_O_2_ are mixed and catalyzed to generate O_2_ to drive ink movement. Visual quantitative detection of SNPs can be realized according to the distance of ink movement. The use of BDNPs to replace the traditional fluorescence reporter eliminates the need for complex fluorescence instruments and can complete quantitative detection of targets against the background of high-concentration DNA, which solves the shortcomings of traditional detection methods (Shao et al., 2019).

**FIGURE 4:**
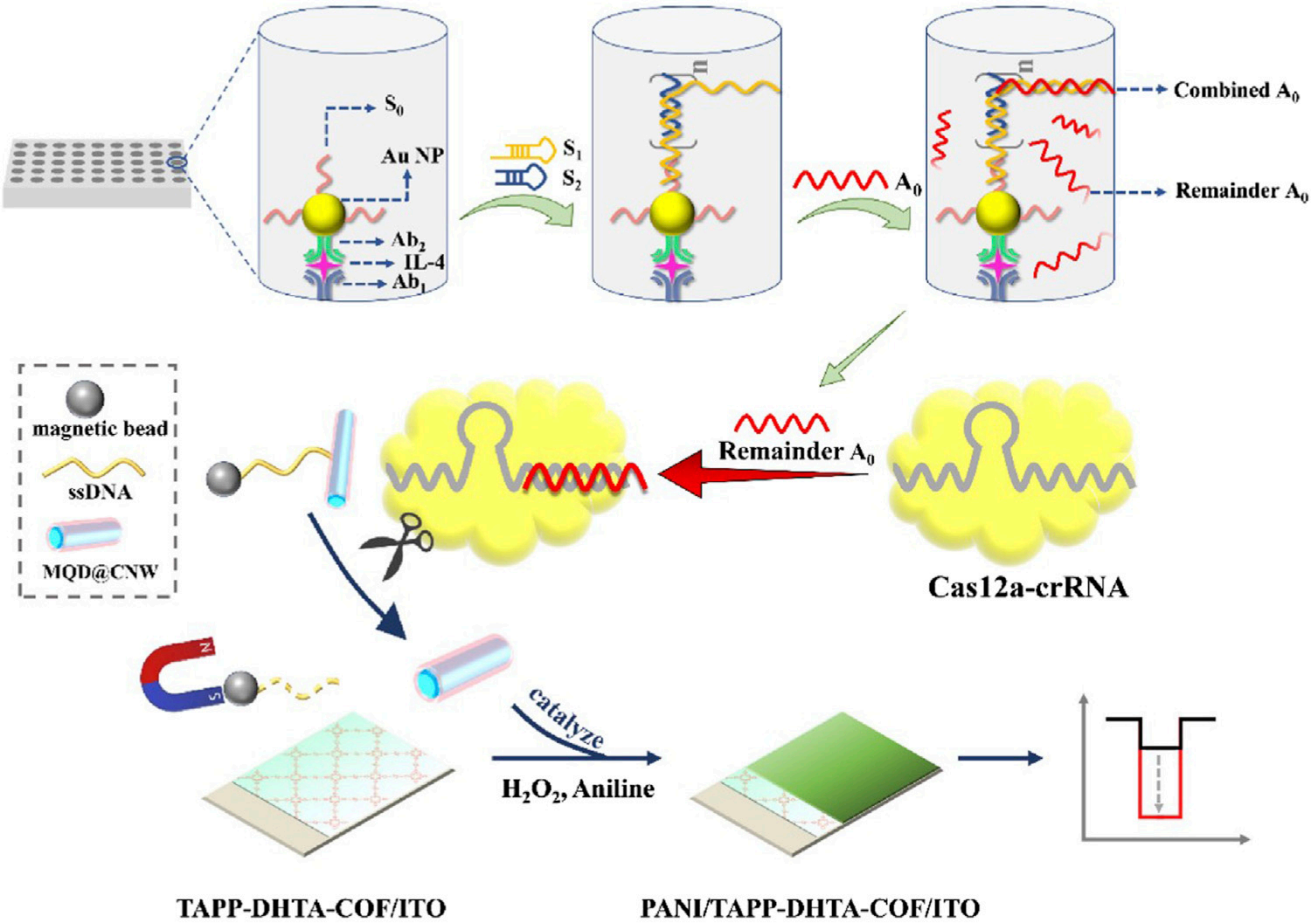
Immunomagnetic bead techniques assist in amplifying the signal of nanozymes. The immuno-HCR-CRISPR/Cas12a-based PEC immunosensor for IL-4. Adapted with permission from Zhang X. et al. (2023), copyright 2023, Elsevier.

Thus, as a kind of simulated enzyme with high catalytic activity, nanozymes can be used as signal amplifiers to assist the CRISPR/Cas12 system in preamplification-free detection. For example, Xu et al. used MnO_2_ nanozymes combined with the CRISPR/Cas12a system to detect Pb^2+^ efficiently and quickly by a colorimetric strategy using immunomagnetic beads as signal enrichment media. MnO_2_ nanorod (MnO_2_ NR) nanozymes linked to magnetic beads via ssDNA have been prepared as colorimetric signal probes in the CRISPR/Cas12a system. Because MnO_2_ NRs have excellent oxidation-like activity, they can oxidize TMB and produce a typical blue product visible to the naked eye. When Pb^2+^ is present, GR5-DNAzyme, which has a high recognition of Pb^2+^, can cleave DNA probes at the rA site, releasing short-length DNA (tDNA). The released tDNA can activate CRISPR/Cas12a trans-cleavage activity, which in turn cleaves the ssDNA linker, separating MnO_2_ NRs from the MB surface to the supernatant, resulting in a color shift that can be observed with the naked eye. This strategy provides a simple, visual and preamplification-free colorimetric method for the detection of Pb^2+^ through the enrichment of magnetic beads and the signal amplification of nanozymes (Table 1) (Xu et al., 2023). Zhang et al. used IMBT as a signal amplification mediator and constructed a cathodic photoelectrochemical (PEC) sensor without preamplification based on the MQD@CNW nanozyme and CRISPR/Cas12a system for IL-4 detection. First, the scholars connected the DNA strand S_0_ onto Au NPs to detect the antibody Ab2, thereby constructing the immune-HCR complex Ab2-Au NP-S_0_. Ab2-Au NP-S_0_ is then confined to a 96-well plate by immune recognition between Ab2 and IL-4. When DNA strands (S1 and S2) are added, the HCR reaction is triggered, forming a long DNA hybridization strand that captures activator DNA A_0_. Subsequently, Cas12a specifically recognizes and binds to the remaining free A_0_ to activate the CRISPR/Cas12a system. The activated CRISPR/Cas12a system further cleaves the long ssDNA ligand between the MQD@CNW nanozyme with peroxisase-like activity and MB through trans-cleavage activity. In the presence of H_2_O_2_, the MQD@CNW releases catalyzed aniline to form polyaniline on the surface of the TPTA-DHTA-COF electrode. This phenomenon results in a significant increase in PEC signals. If IL-4 is present in the sample, the free DNA A_0_ is reduced; thus, the specifically activated Cas12a is reduced, and the cleavage of the MQD@CNW-ssDNA-MB complex is increased. Moreover, under the action of magnetic field separation, the MQD@CNW nanozyme that can catalyze aniline is reduced; therefore, the signal is significantly weakened. The IL-4 contents in the samples are detected by the significant change in the PEC signal. Therefore, based on the application of immunomagnetic beads and the specific immune recognition and multiple signal amplification of immune-HCR-CRISPR/Cas12a, the interference of background signals can be eliminated, and a simple and reliable IL-4 immunoassay can be achieved (Figure 4; Table 1) (Zhang X. et al., 2023b).

### 4.4 Dopamine-modified probe-assisted nanozymes inspired hypersensitive fluorescence signals

In the preparation of fluorescent probes, dopamine is frequently employed for probe modification to enhance both their specificity and sensitivity. Dopamine monomers can undergo oxidative self-polymerization, resulting in the formation of a polymer known as polydopamine (PDA). PDA NPs serve as broad-spectrum fluorescence quenchers and possess robust quenching capabilities. PDA closely mimics the composition and structure of natural melanin, often exhibiting characteristics akin to its natural counterpart. Notably, PDA exhibits exceedingly weak intrinsic fluorescence and can quench fluorescence through mechanisms such as fluorescence resonance energy transfer (FRET) and photoinduced electron transfer (PET). Consequently, dopamine can be utilized for the modification of probes and fluorescent probes can be directly synthesized through the oxidative self-polymerization of dopamine. Due to its exceptional fluorescence stability, biocompatibility, and water solubility, dopamine plays a pivotal role in biomarker detection. Numerous studies employ dopamine-modified fluorescent probes, where the disruption of the linkage between dopamine and the fluorescent probe results in a fluorescence quenching effect followed by fluorescence recovery. This transition serves as a fluorescence signal indicator, switching from a signal-off state to a signal-on state, enabling the rapid and highly sensitive detection of target genes (Wang L. et al., 2020a).

Based on the remarkable catalytic activity of nanozymes, dual-responsive fluorescence sensors based on nanozyme-dopamine with dual-phase ratios can significantly amplify fluorescence signals (Kong et al., 2023). When integrated with the CRISPR/Cas system, these sensors exhibit heightened sensitivity and precision in the detection of target DNA/RNA.

**FIGURE 5:**
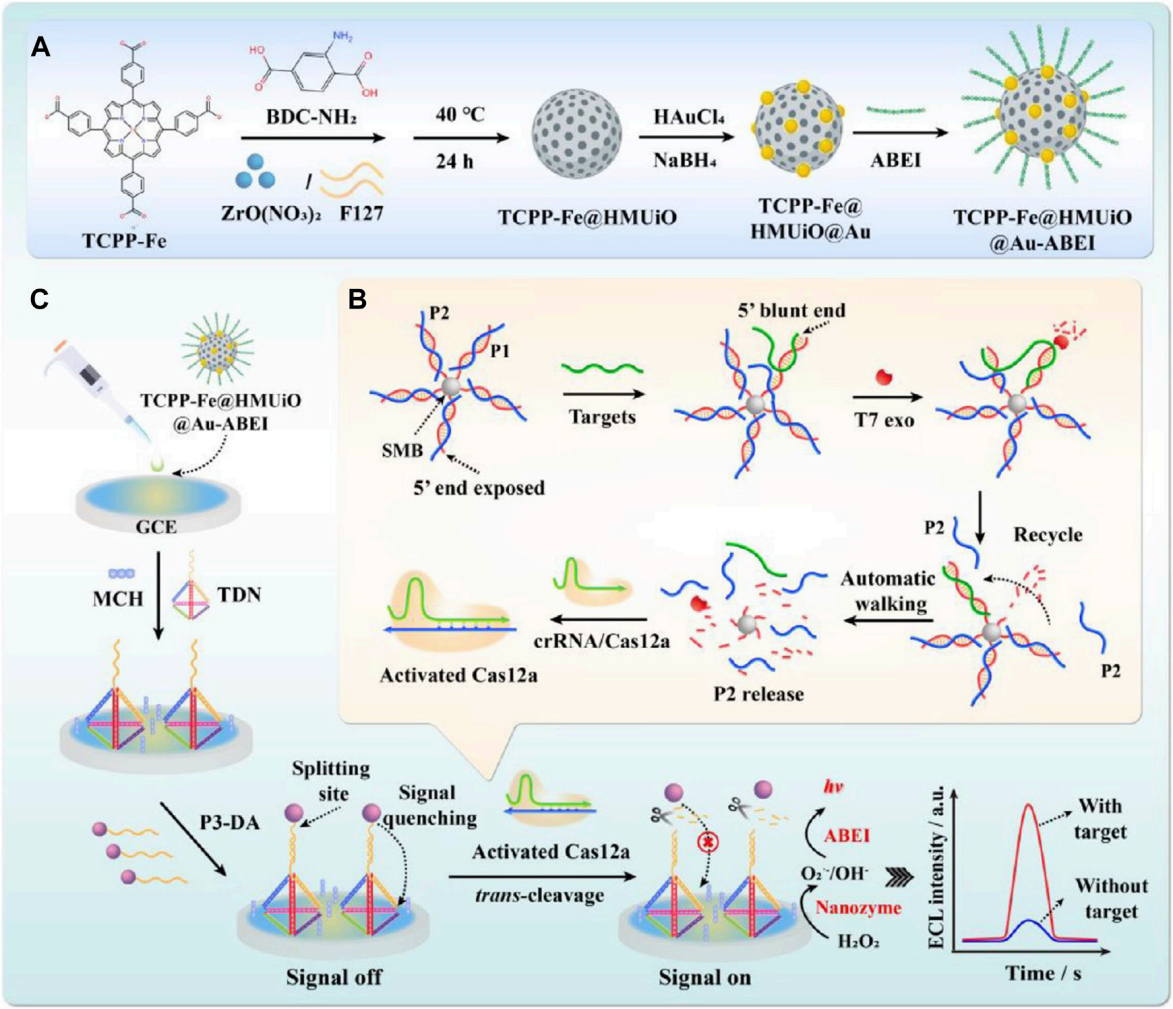
Dopamine-modified probe-assisted nanozymes inspired hypersensitive fluorescence signals. Construction of the mesoporous nanozyme-enhanced DNA Tetrahedron electrochemiluminescent biosensor combined with 3D walking nanomotor-mediated CRISPR/Cas12a. **(A)** Synthesized procedure of the nanoemitters. **(B)** Construction of the 3D walking nanomotor-mediated CRISPR/Cas12a strategy. **(C)** Constructed process of the EC bLiosensor. Adapted with permission from Shen et al. (2023), copyright 2023, American Chemical Society.

For example, Shen et al. designed and developed an amplification-free ultrasensitive miRNA detection biosensor by combining dopamine-modified DNA probes with nanozymes to display detection results through on-off fluorescence signals. The scholars mediated CRISPR/Cas12a activation using an exonuclease with highly efficient driving properties combined with the TCPP-Fe@HMUiO@Au-ABEI nanozyme modified by tetrahedral DNA nanostructures (TDNs) for fluorescence signal amplification. An ultrasensitive electrochemical luminescence (ECL) biosensor for the detection of exomiR-155 is constructed. The design strategy of this biosensor is that the 3′-end of the biotin-modified dsDNA probe (P1/P2) is first fixed on a streptavidin-labeled magnetic bead (SMB). The presence of exomiRNA-155 can shift the P2 chain to form exomiRNA-155/P1 with a blunt 5′-end. When exomiRNA-155 binds to P1, due to the splicing of the T7 exo, the T7 exo can enzymatically degrade P1, releasing the targets in the 5′→3′ direction. The released targets move autonomously along a predesigned DNA track, resulting in the release of large amounts of P2. P2 then activates Cas12a in response to crRNA, thereby activating its trans-cleavage ability, which can degrade the dopamine-modified ssDNA strand (P3-DA). In the absence of the target exomiRNA-155, the dopamine-modified P3 chain (P3-DA), which is not cleaved, is hybridized with TDNs to suppress the original ECL response (signal off state). In the presence of exomiRNA-155, CRISPR/Cas12a cleavage of P3-DA releases DA, promoting TCPP-Fe@HMUiO@Au nanozymes with excellent catalytic properties to excite ABEI and obtain a strong ECL response (signal on state). Thus, the target exomiR-155 can be effectively converted into an amplified biological signal, improving the sensitivity and specificity of detection. Finally, the detection limit obtained is 273.20 aM with a range of 1.0 fM to 1.0 nM. The biosensor can ultrasensitively detect trace amounts of miRNA, providing a promising tool for early clinical diagnosis (Figure 5; Table 1) (Shen et al., 2023). Liu et al. (2022) constructed a novel self-electrochemical luminescence (ECL) biosensor based on PdCuBP@luminol nanoemitters with excellent oxidase-like catalytic activity combined with the CRISPR/Cas12a system for high-sensitivity detection of COX III genes. The scholars used dopamine-modified thiohairpin DNA (hpDNA-DA) as a DNA probe that acts as a fluorescence quencher and turns off the signal. When the COX III gene is present, CRISPR/Cas12a trans-cleavage activity is activated to cleave the hpDNA-DA probe and release DA, thereby restoring the ECL signal. Moreover, the hpDNA probe can further promote the oxidase-like catalytic activity of PdCuBP@luminol nanozymes, accelerate the conversion of dissolved O_2_ to ROS, and enhance the ECL signal. The biosensors with PdCuBP@luminol nanoemitters combined with the CRISPR/Cas12a system show excellent analytical performance for COX III gene detection with an amplification-free reaction. Benefiting from the efficient ECL emission of the nanozymes and the CRISPR/Cas12a-mediated interfacial cleavage of the DA quencher, the developed ECL biosensor has a high sensitivity to COX III with a detection limit as low as 0.18 pM. Therefore, this strategy provides broad prospects for nucleic acid detection in the field of clinical diagnosis (Table 1) (Liu et al., 2022).

## 5 Conclusion

In the field of biomarker detection, CRISPR-based technologies continue to be explored, with significant potential applications in the field of *in vitro* diagnostics (van Dongen et al., 2020). The detection strategies of the CRISPR/Cas system are based on the binding activity, cis-cleavage activity, and trans-cleavage activity of Cas effector molecules. Combined with various signal technologies, such as electrochemical, electrochemical, fluorescent, and colorimetric methods, CRISPR-based detection has demonstrated the ability to achieve high sensitivity in target analyte detection (Tian et al., 2022; Wang et al., 2022; Li Z. et al., 2023b). While CRISPR/Cas-based detection technologies have made considerable progress, they still present relatively high costs and limited multichannel detection capabilities.

Nanozymes play a vital role in enhancing the sensitivity of CRISPR-based detection, enabling visual and rapid CRISPR testing. Utilizing the remarkable enzyme-like catalytic activity of nanozymes to amplify electrochemical and optical signals appears to be a promising strategy, including electrochemical and colorimetric signal sensors (Lee et al., 2022; Mou et al., 2022). The use of nanozymes to amplify detection signals results in increasingly sensitive and accurate outcomes. Nanozymes, as a category of nanomaterials with enzyme-like catalytic activities, combine the advantages of biological enzymes and nanomaterials, displaying high catalytic performance and exceptional stability (Huang et al., 2019; Singh et al., 2023). Relative to natural enzymes, nanozymes exhibit various advantages, including enzyme-like reaction kinetics, high catalytic efficiency, and tolerance to environmental variations. Moreover, nanozymes are highly customizable, contributing to their superior catalytic activity relative to natural enzymes. As a result, nanozymes enhance the sensitivity of CRISPR/Cas detection systems by offering stability and high catalytic activity, thereby improving the detection process. Due to their unique enzyme-like properties and the possibility of achieving colorimetric reactions, nanozymes have gained significant attention for the sensitive detection of biomolecules using colorimetry, fluorescence, and electrochemical methods under complex physiological conditions. Thus, nanozymes, as signal markers in biomolecular detection, can overcome many limitations associated with natural enzymes. Based on the limitations of natural enzymes in visual detection, nanozymes exhibit several advantages, such as high stability and efficient catalysis. In complex reactions within the CRISPR/Cas system, nanozymes can circumvent the deficiencies of native enzymes, as they are less stable and vulnerable to the effects of enzyme activity. Through the assistance of nanozymes in ratiometric fluorescence detection, nanozyme-based fluorescence assays can avoid the interference of background fluorescence, significantly enhancing sensitivity and accuracy. In certain designs, ratiometric fluorescence probes with nanozyme assistance can exhibit noticeable color changes, enabling visual detection. Through the modifiability of nanozymes, they can be connected with specific probes. In reactions, cleaved nanozymes can further amplify the detection signal, providing an advantage over other fluorescence probes and biotin in CRISPR/Cas system applications. Additionally, modification with negatively charged polymers can enhance nanozyme catalytic activity. For instance, modifying HCR products increases the affinity of nanozymes for peroxidase substrates, thereby greatly improving detection sensitivity. Nanozyme-assisted CRISPR detection strategies have been developed for the detection of DNA/RNA, achieving LODs in the picomolar (pM) range in a short period.

In addition to strategies that enhance the catalytic activity of nanozymes, there is a need for additional methods to achieve visual signal amplification strategies without the requirement for preamplification. For example, padlock probes can be employed to initiate intracellular rolling circle amplification (RCA) reactions without the need for preamplification. By adapting ligand/DNA-modified nanozymes to enhance the catalytic activity of nanozymes, signal amplification can be achieved within the CRISPR/Cas system. Furthermore, the capture and enrichment of target analytes can be accomplished using immunomagnetic bead separation technology combined with the action of colorimetric signal probes, efficiently separating target analytes from the system and assisting in the signal amplification of nanozymes while eliminating external environmental interference. This technology provides a simple, visual, and preamplification-free colorimetric method for target analyte detection. Moreover, dopamine-modified probes can assist in nanozyme-mediated ultrasensitive fluorescence signal amplification. Based on the outstanding enzyme-like catalytic activity of nanozymes, dual-responsive fluorescence sensors based on nanozyme-dopamine with dual-phase ratios can significantly amplify fluorescence signals. When combined with the CRISPR/Cas system, these sensors exhibit relatively high sensitivity and accuracy in the detection of target DNA/RNA.

In the future, there should be efforts to develop CRISPR-based detection systems assisted by nanozymes with short detection times and simple data outputs, expanding their potential applications in on-site testing.
